# Enhancement of Magnetic and Dielectric Properties of Ni_0.25_Cu_0.25_Zn_0.50_Fe_2_O_4_ Magnetic Nanoparticles through Non-Thermal Microwave Plasma Treatment for High-Frequency and Energy Storage Applications

**DOI:** 10.3390/ma15196890

**Published:** 2022-10-04

**Authors:** Muhammad Adnan Munir, Muhammad Yasin Naz, Shazia Shukrullah, Muhammad Tamoor Ansar, Muhammad Umar Farooq, Muhammad Irfan, Salim Nasar Faraj Mursal, Stanislaw Legutko, Jana Petrů, Marek Pagáč

**Affiliations:** 1 Department of Physics, University of Agriculture Faisalabad, Faisalabad 38040, Pakistan; 2 Centre of Excellence in Solid State Physics, University of the Punjab, Lahore 54590, Pakistan; 3 State Key Laboratory of Chemical Engineering, East China University of Science and Technology, 130 Meilong Road, Shanghai 200237, China; 4 Electrical Engineering Department, College of Engineering, Najran University, Najran 61441, Saudi Arabia; 5 Faculty of Mechanical Engineering, Poznan University of Technology, 60-965 Poznan, Poland; 6 Department of Machining, Assembly and Engineering Metrology, Mechanical Engineering Faculty, VŠB-Technical University of Ostrava, 17, Listopadu 2172/15, 70800 Ostrava, Czech Republic; 7 FME, Department of Machining, Assembly and Engineering Metrology, VSB Technical University of Ostrava, 17. listopadu 2172/15, 70800 Ostrava, Czech Republic

**Keywords:** ferrite nanoparticles, non-thermal plasma, dielectric properties, magnetization, conductivity, energy storage

## Abstract

Spinel ferrites are widely investigated for their widespread applications in high-frequency and energy storage devices. This work focuses on enhancing the magnetic and dielectric properties of Ni_0.25_Cu_0.25_Zn_0.50_ ferrite series through non-thermal microwave plasma exposure under low-pressure conditions. A series of Ni_0.25_Cu_0.25_Zn_0.50_ ferrites was produced using a facile sol–gel auto-ignition approach. The post-synthesis plasma treatment was given in a low-pressure chamber by sustaining oxygen plasma with a microwave source. The structural formation of control and plasma-modified ferrites was investigated through X-ray diffraction analysis, which confirmed the formation of the *fcc* cubical structure of all samples. The plasma treatment did not affect crystallize size but significantly altered the surface porosity. The surface porosity increased after plasma treatment and average crystallite size was measured as about ~49.13 nm. Morphological studies confirmed changes in surface morphology and reduction in particle size on plasma exposure. The saturation magnetization of plasma-exposed ferrites was roughly 65% higher than the control. The saturation magnetization, remnant magnetization, and coercivity of plasma-exposed ferrites were calculated as 74.46 *emu/g*, 26.35 *emu/g,* and 1040 Oe, respectively. Dielectric characteristics revealed a better response of plasma-exposed ferrites to electromagnetic waves than control. These findings suggest that the plasma-exposed ferrites are good candidates for constructing high-frequency devices.

## 1. Introduction

Ferrites are ceramic materials containing a large amount of iron oxide (Fe_2_O_4_) mixed with metallic elements such as zinc (Zn), cobalt (Co), nickel (Ni), and manganese (Mn) in minuscule proportions. Iron oxide possesses ferrimagnetic and electrically non-conducting behavior [1]. Ferrites are categorized into two types, i.e., soft and hard ferrites. Soft ferrites have the potential to magnetize and demagnetize easily, while hard ferrites, due to greater *H_c_* and *M_r_,* are difficult to magnetize and demagnetize [2,3]. Among soft ferrites, spinel ferrites are presented by MFe_2_O_4_ where *M* reveals divalent ions, generally known as Mn^2+^, Zn^2+^, Fe^2+,^ and Co^2+^. Excellent magnetic and electrical and microwave characteristics make spinel ferrites a strong candidate for numerous applications [4], such as high-frequency applications [5], recording heads [6], antimicrobial agents [7], microwave absorbers [8], and catalysts [9]. With the advancement of electronic devices regarding compact design, multifunctionality, and miniaturization, high-power inductors are attracting the attention of researchers for new developments in electronics. Multilayer chip inductors (MLCIs) are extensively utilized in the miniaturization of energy storage appliances. NiCuZn ferrite has been widely tested as a substrate magnetic material for MLCIs due to its high resistance and exceptional magnetic characteristics in high-frequency ranges [10]. MLCIs are key components in video cameras, smartphones, and notebook computers, where improved magnetic attributes of nanoferrites are very important for efficient MLCIs to minimize ferrite volume in the chip. So, with the advancement of critical component downsizing, the formulation of NiCuZn ferrite with excellent characteristics has sparked broad interest [11]. To achieve the technological merits of miniaturizing dense cores for high frequencies, more research on NiCuZn ferrites has been conducted to investigate the physical phenomenon responsible for magnetic modifications. Chu et al. [12] investigated the role of Mn^2+^ doping in improving magnetic characteristics of spinel NiCuZn ferrites. Experimental results revealed that saturation magnetization reduced from 67 to 62 *emu/g* when Mn^2+^ content increased up to 0.03%. With a rise in sintering temperature, both remanence and saturation magnetization increased while coercivity was decreased. Mn^2+^ addition cause an increase in permeability with a drop in resonance frequency. Peng et al. [13] synthesized NiCuZn ferrite via a co-precipitation method with ultrasonic assistance and analyzed the influence of temperature and ultrasonic radiation on magnetic characteristics and microstructures of nanoferrites. The best magnetic properties were obtained at room temperature (RT) with assistance of 60W ultrasonic power. *M-H* investigation revealed that with the same ultrasonic power NiCuZn ferrite exhibited maximum saturation magnetization and coercivity, but at a 50 °C reaction temperature ferrites exhibited ferromagnetic behavior. It was concluded that ultrasound has a progressive effect on the microstructure and magnetic attributes of ferrite NPs and this technique also has the potential for the controlled preparation of ferrites at an industrial scale. Sujatha and colleagues investigated how the sintering temperature affected the electromagnetic properties of NiCuZn ferrite. Experimental findings revealed that temperature directly affects material features and excellent magnetic and dielectric properties were exhibited with increased sintering temperature [14]. Researchers have adopted numerous routes such as the sol–gel auto-combustion technique [15], solid-state route [16], hydrothermal method [17], co-precipitation [18], and egg white technique [19] for the synthesis of NiCuZn ferrite. Magneto-electric characteristics can be tuned using different additives such as Bi_2_O_3_, V_2_O_5_, and Cu [20,21,22]. Microwave heating has also been utilized to synthesize NiCuZn ferrite due to several benefits over other techniques such as minimum reaction time, rapid and selective heating, and direct interactions of dipoles and charges in pristine specimens [23]. Post-synthesis plasma processing of nanoparticles is another approach adopted to improve the opto-magnetic characteristics of materials [24]. Microwave plasma is non-thermal plasma generated by an electrode system under low pressure and a high-power microwave source. Microwave radiation generates electrons, ions, and high-energy neutral species which directly react with the target substance and alter the texture and characteristics of that material [25]. In fact, the plasma-assisted process generates free radicals, which produce crystalline vacancies on inorganic substances, altering the electronic distribution and supplying appropriate active sites [26,27].

To date, no detailed research has been reported on improving the magnetic and dielectric characteristics of NiCuZn ferrite using post-synthesis plasma treatment. Therefore, in the current research, our prime motivation is to probe the impact of microwave plasma treatment on NiCuZn ferrites for high-frequency devices. In this research, NiCuZn ferrite is successfully fabricated by the sol–gel auto-ignition method. The post-synthesis impact of plasma exposure is tested for ferroelectric, dielectric, and magnetic attributes of spinel ferrite, which reveals substantial enrichment in magnetic and dielectric characteristics.

## 2. Experimental Work

### 2.1. Synthesis of NiCuZn Ferrite

**Figure 1:**
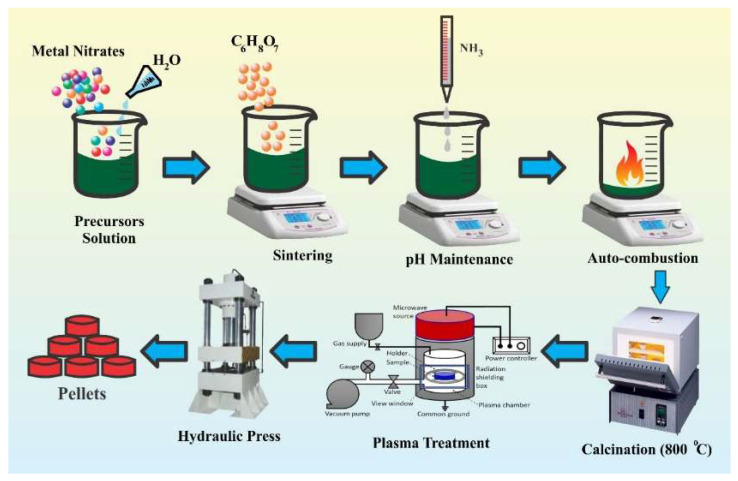
Schematic illustration of synthesis and plasma treatment of ferrites.

Ni_0.25_Cu_0.25_Zn_0.50_ ferrite samples were fabricated via the sol–gel auto-combustion method. Estimated quantities of metal nitrates, i.e., zinc nitrate (Zn (NO_3_)_2_ ≥ 98.5%], nickel nitrate (Ni (NO_3_)_2_ ≥ 99.98%], iron nitrate (Fe (NO_3_)_3_ ≥ 98.5%], and copper nitrate (Cu (NO_3_)_2_ 99.95%], were used as precursors. Citric acid C_6_H_8_O_7_ was utilized as fuel for combustion and liquid ammonia NH_3_ was used to maintain the pH of the solution at 7. In the first step, metal nitrates were dissolved individually in DI water. Then, an aqueous solution of citric acid was introduced to the metal nitrate solution, keeping the nitrate to citrate proportion at 1:1. Liquid ammonia was added to this solution dropwise under continuous stirring. After adjusting the pH of the nitrate citrate solution, constant heat at 90 °C was supplied during the hydrolysis of the solution. Continuous heating and stirring resulted in the formation of xerogel, which further burned due to the exothermic process generating flames in the beaker. The final product was black ash-like powder. The obtained fluffy powder was dried at 100 °C and then calcined at 800 °C for 4 h. Three samples were fabricated using 30, 40, and 45 mL of DI water and named as UT30, UT40, and UT45, respectively. Finally, one half was taken from all prepared NiCuZn ferrite samples and was exposed to microwave plasma. Plasma-treated samples with different DI water concentrations were named as PT30, PT40, and PT45, respectively. All synthesized untreated and plasma-treated samples were converted into a toroid using a hydraulic press. Schematic of synthesis and plasma treatment process is provided in Figure 1.

### 2.2. Non-Thermal Plasma Treatment

**Figure 2:**
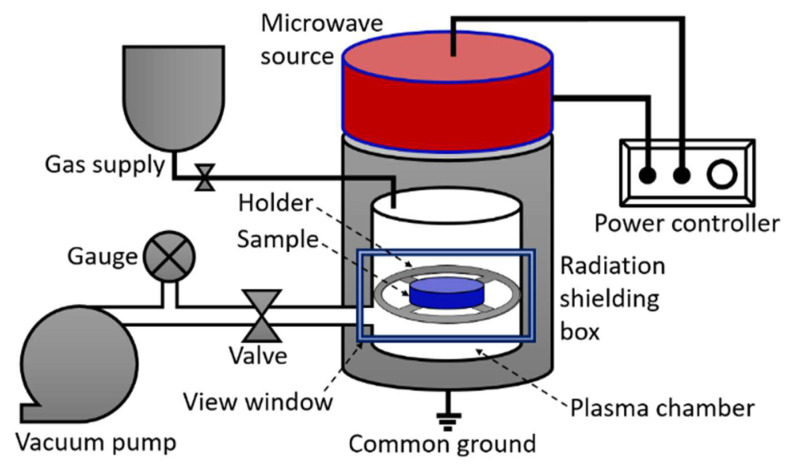
Illustration of plasma processing setup.

Non-thermal plasma generated by microwaves with a 900 W power source was used to treat ferrites. Figure 2 displays the plasma treatment setup comprise of different components. First of all, specimens were placed in a sample holder and the sample holder was further placed in a shielding box to prevent radiation leakage. The chamber was fully evacuated using a vacuum pump which created low pressure in the chamber. After creating the required pressure, oxygen gas was supplied as a plasma precursor. The precursor gas was provided to the fully evacuated chamber. The plasma was made by using a microwave source that breaks the gas atoms into free electrons and highly energetic ions, collectively called plasma. A gradual increase in microwave power resulted in plasma production at a certain power level and then continuous production of plasma started in the chamber at a power of 1200 W. Each pristine specimen was exposed to plasma for half an hour, which directly interacted with specimens and caused surface modifications and changes in magnetic and dielectric characteristics of ferrites. Both pristine and processed ferrite samples were pressed into pellets with a hydraulic press at an external force of 40 k N.

### 2.3. Characterization

A Bruker D8 X-ray diffractometer was used to determine the phases of the fabricated ferrite specimens. A radiation source with a wavelength of 1.54 Å was used in this characterization. The morphology was probed using a NovaNano SEM 450 FESEM instrument. Magnetic characteristics of pristine and plasma-treated ferrites were measured using a Lakeshore-7410 VSM instrument (vibrating sample magnetometer). The dielectric behavior of specimens was analyzed by employing a Wayne-Kerr 6500B precision analyzer. The ferroelectric characteristics of plasma-treated and pristine samples were examined using a multiferroic tester from Radiant Technology Inc (Albuquerque, NM, USA).

## 3. Results and Discussion

### 3.1. Phase Analysis

**Figure 3:**
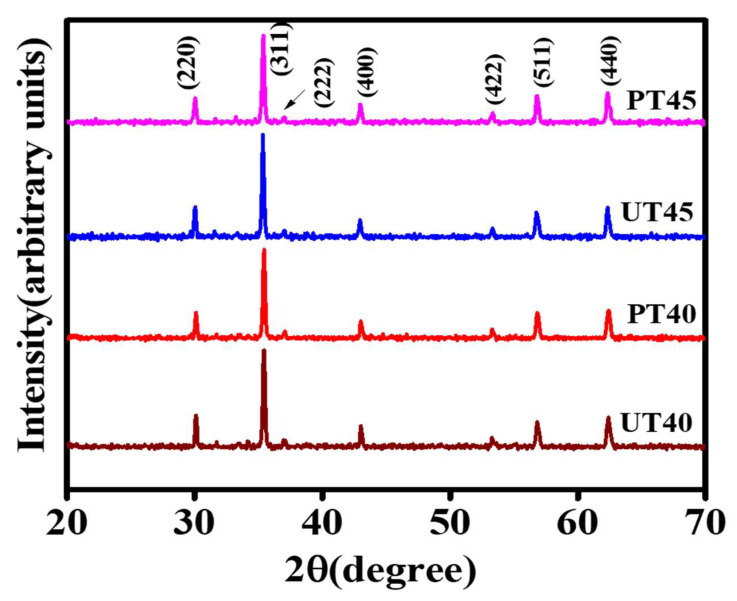
Indexed XRD spectra of pristine and plasma-processed NiCuZn ferrite.

X-ray diffraction was employed to probe crystal structure, phase purity, and other structural parameters of ferrite specimens. The synthesis process and annealing temperature have a significant role in determining the crystal structures of nanoparticles [28]. XRD analysis of Ni_0.25_Cu_0.25_Zn_0.50_ was conducted within the 2θ range of 20°–80° to probe the structural and phase compositions of both pristine and plasma-processed NiCuZn ferrite samples calcined at 800 °C. The XRD peak indexing was executed using a technique elucidated by Cullity [29]. XRD intensity peaks were reported at 2θ of 30.03°, 35.35°, 36.96°, 42.94°, 53.29°, 56.79°, 62.31°, and 73.76°, which are attributed to (220), (311), (222), (400), (422), (511), and (440) *hkl* planes, respectively, mentioned in Figure 3. These *hkl* planes indicated the growth of pure *fcc* spinel structures of NiCuZn ferrite and obtained peaks agreed with the previously reported literature [30]. Obtained XRD patterns also endorsed the statement that the sol–gel auto-ignition technique is favorable for synthesizing spinel ferrites using described conditions [31]. 

The magnificent crystallinity of specimens was palpable, as shown by the sharp peak associated with the (311) plane and FWHM related to the development of crystallites [32]. Table 1 shows different parameters, including crystallite size, lattice parameters, X-ray density, bulk density, and porosity. The lattice parameters of all specimens were estimated by applying Equation (1) [33]:

(1) $$a=\frac{\lambda }{sin\theta }\;\sqrt{(h^{2}+k^{2}+l^{2}})$$

A simple relation (V = a^3^) was used to find out the unit cell volume [33], while grain size was obtained from Scherrer’s formula in Equation (2) by using the (311) characteristic peak for all specimens [29].

(2) $$D_{hkl}=\frac{K\lambda }{\beta COS\theta }$$

Here, θ is the diffraction angle, *β* denotes FWHM, and *K* is the shape factor. The X-ray is denoted by λ, which is typically 1.54 Å. Furthermore, X-ray density ρx was evaluated by applying Equation (3) [33].

(3) $$\rho x=\frac{ZM}{VN_{A}}$$

Here, N_A_ is Avogadro’s no., *V* is unit cell volume, *M* defines molecular weight, and *Z* reveals the no. of formula units existing in the unit cell. The bulk density of pressed samples was estimated using Equation (4) [34].

(4) $$\rho_{B}=\frac{m}{v}$$

Here, m is the mass of pellets prepared using a hydraulic press while v is the volume of pellets calculated by using the relation given in Equation (5).

*V* = *πr*^2^*h* (5)

In this relation, *r* is the pellet’s radius, while *h* presents the pellet’s height. The porosity of the synthesized specimens was also calculated using the relation between X-ray density and bulk density presented in Equation (6) [31].

(6) $$\mathrm{Porosity}=\left(1-\frac{\rho B}{\rho x}\right)\times 100\%$$

All calculated parameters mentioned in Table 1 revealed that plasma exposure did not alter the phase and structure formation of spinel ferrites.

### 3.2. Microstructural Analysis

**Figure 4:**
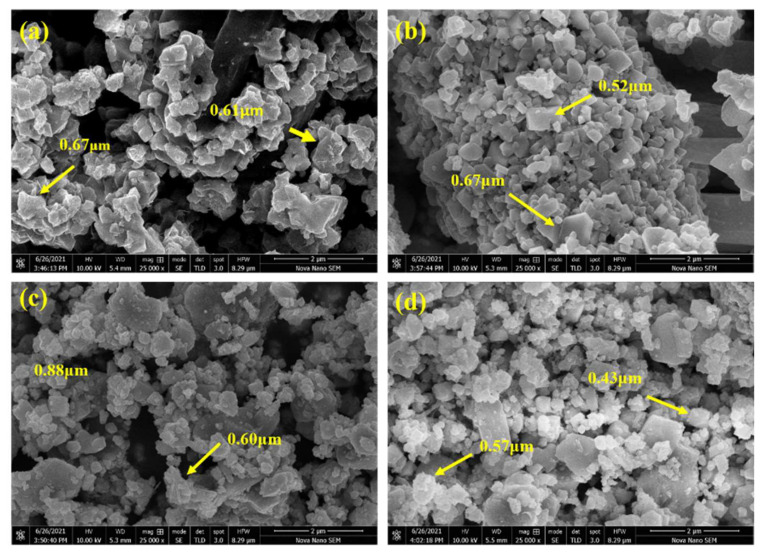
SEM micrograph of pristine UT40, UT45 4 (**a**,**c**), and plasma-treated PT40, PT45 4 (**b**,**d**) specimens.

**Figure 5:**
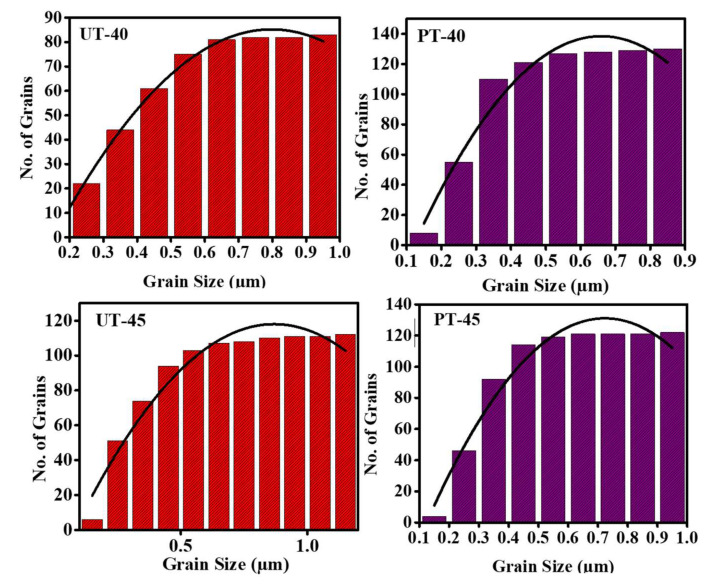
A statistical representation of grain distribution for pristine and plasma−treated ferrite.

The morphological representation and statistical grain distribution of pristine and processed ferrite nanoparticles are given in Figure 4 and Figure 5. It is conspicuous from Figure 4a,c that untreated NiCuZn ferrites exhibited dense and homogeneous morphologies with non-uniform grain distribution. Nanoparticles exhibited an almost spherical shape with a cluster-like appearance. Micrographs also show that pristine NiCuZn ferrites demonstrated fuzzy grain boundaries with agglomeration among nanoparticles. Generally, agglomeration appeared because of high annealing temperature magnetic dipole–dipole interactions among the nanoparticles [35]. When the agglomeration phenomena dominated, the grains clumped together, reducing porous behavior. Figure 4b,d shows SEM micrographs of plasma-treated specimens. It is conspicuous that plasma exposure significantly affected the morphology of ferrite specimens. Plasma treatment alters not only the shape but also the size of grains. A decrease in grain size caused a decline in agglomeration due to plasma treatment which was also reported before [36]. Some grains exhibited a cubical shape, while most grains exhibited a spherical shape with well-defined grain boundaries. Changes in morphology and grain shape indicate that plasma treatment is a very useful technique in surface modifications. These changes in surface morphology correspond to the plasma etching effect [37]. A noticeable transformation in shape and size was reported, which are influential factors for determining the magnetic and dielectric features of spinel ferrite [38]. The majority of grains showed sizes ranging from 0.6 to 1 μm in the case of pristine ferrite specimens, as shown by histograms in Figure 5.

### 3.3. Magnetic Features

**Figure 6:**
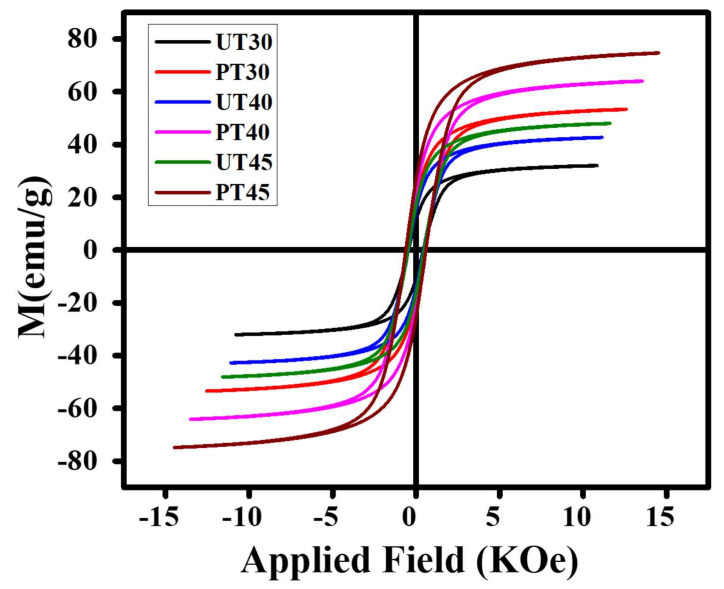
Variation in saturation magnetization, coercivity and remanence magnetization with plasma treatment.

VSM investigation was accomplished to examine the magnetic attributes such as *M_s_, H_c_, M_r_*, etc. The magnetic characteristics of spinel ferrites are affected by various factors, including synthesis route, cation distribution on tetrahedral and octahedral sites, and crystallite size, as presented in Figure 5 [30]. Magnetic characteristics of ferrite nanoparticles can be modified using different doping ions or changing the synthesis technique and calcination temperature [39,40]. Hysteresis loops of both untreated and plasma-treated ferrites were plotted using applied field and corresponding magnetization. Values of magnetic parameters were calculated using hysteresis loop and are presented in Table 2. It is evident from Figure 6 that plasma treatment effectually increased *M_s_*, *H_c_*, and *M_r_*. A more than 60% increase in *M_s_* was observed for plasma-treated ferrite specimens and the highest value was 74.46 *emu/g,* which agreed with that previously reported for La-doped NiCuZn ferrites [41]. Saturation magnetization reported in this research is the highest magnetization observed for pure NiCuZn ferrite and also greater than for ferrite mixed with additives, i.e., Cu, Tb, Co, and Bi_2_O_3_-Nb_2_O_5_ [22]. That escalation in saturation magnetization is associated with direct contact of ferrites specimens with highly energetic microwave plasma [42]. 

When ferrite specimens are directly exposed to microwave plasma, the movement of magnetic dipoles significantly improved due to rising temperature, which boosts saturation magnetization [43]. An increase in coercivity and remanence can be associated with surface effects generated during plasma treatment [44]. Analysis of magnetic characteristics endorsed the statement that microwave plasma treatment is an efficient technique used for the modification of ferrites and it can be valuable in the latest applications.

### 3.4. Ferroelectric Properties 

**Figure 7:**
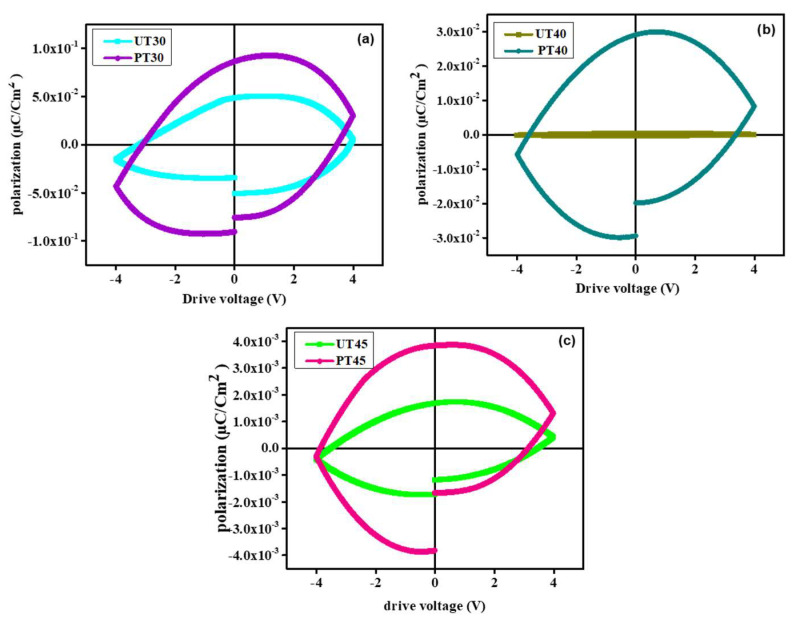
Polarization−electric field profiles of UT30, PT30 (**a**) UT40, PT40 (**b**), and UT45, PT45 (**c**).

The ferroelectric characteristics of pristine and plasma-treated NiCuZn ferrites were analyzed via *P-E* loops by plotting curves using polarization and electric field at a 50 Hz frequency and RT and are reflected in Figure 7 [45]. From the formation of *P-E* loops, it is evident that both pristine and plasma-treated specimens exhibited weak ferroelectric characteristics [46]. The remnant polarization (*P_R_*), maximum polarization (*P_m_*), and coercive field (*E_c_*) were significantly affected by plasma exposure. Untreated ferrite specimens exhibited narrow *P-E* loops, while plasma-treated specimens revealed oval-like *P-E* loops indicating deterioration in ferroelectric attributes for plasma-treated ferrites [47]. The development of wider *P-E* loops revealed substantial leakage current, which is anticipated due to the existence of conducting ferrite phase [48]. The excellent ferroelectric characteristics of untreated ferrite specimens can be related to the restricted mobility of ferroelectric phase domains [49]. Overall analysis validated that plasma treatment significantly reduced the ferroelectric properties of ferrite specimens.

### 3.5. Dielectric Characteristics and Complex Impedance Parameters

Currently, the world is facing environmental and energy challenges. Researchers are working diligently to design efficient energy storage devices with lower environmental hazards [50]. Dielectric substances can be utilized for numerous applications, such as enhancing the performance of capacitors, semiconductors, and other energy storage systems. The dielectric attributes can be probed by using real and imaginary components of permeability (ε′ and ε″), loss tangent (tanδ), and ac-conductivity (σ_ac_) [51]. The dielectric analysis of ferrites provides evidence about the nature of charge carriers and the conduction process accruing in ferrites. The dielectric behavior of magnetic materials is generally influenced by factors such as cation distribution, synthesis technique, temperature, applied field, grain size, and composition of the material [52].

Temperature and frequency dependency has a significant impact on the dielectric characteristics. To examine the dielectric response, the specimens in a pellet shape were placed between a couple of copper electrodes. Resistances and capacitances of ferrite-based specimens were measured using an *ac* signal in the frequency range of 20Hz to 20MHz. The magnitude of *ε*′ of a substance is considered the amount of energy that can be stored in any substance. This energy can be estimated by using the following relation [53]:

ε′ = C_p_d/Aε_o_. (7)

**Figure 8:**
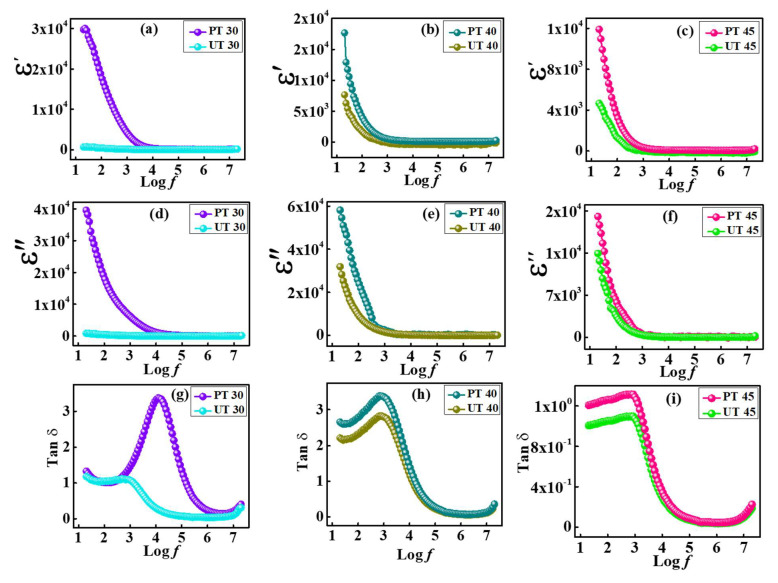
Comparison study of variation in (**a**–**c**) real permittivity, (**d**–**f**) imaginary permittivity, and (**g**–**i**) tangent loss for plasma-treated and untreated samples.

In this equation ε’ represents the dielectric permittivity, ε_o_ denotes the permittivity of free space (8.85 × 10^−12^ F/m), C_p_ represents the parallel capacitance of the specimens, *d* is the width of the sample pellet, and *A* indicates the area of the pellet. Figure 8a–f reveal the typical behavior of ε’ and ε″ while input frequency is supplied at RT. Both pristine and plasma-processed ferrite specimens showed a steady decrease in ε′ with increasing frequency. In the low-frequency zone, ε′ exhibited high values while an upsurge in frequency caused a decline in ε′, providing a consistent linear trend for pristine and plasma-treated samples. The depicted trend is associated with typical ferrite behavior, which appears because of enormously fast fluctuations of the provided ac field [54]. Furthermore, multiple polarization processes appear when ac is supplied, including atomic, electronic, space charge, and orientation polarization. In the low-frequency zone, all mentioned polarization processes support the net mechanism, which provides large values of ε’. Increase in frequency manifests as dominating behavior of atomic and electronic polarization while reducing the orientation and space charge polarization. For the polarization process, the degree of mobility of charge carriers is more prominent, which can be estimated as relaxation time. The dipoles start to align themselves spontaneously when the exterior ac field is supplied. The direction of dipoles remains the same as the applied field and the time consumed during the alignment of charges is termed τ, which can be estimated by using Equation [55]:

τ = 1/(2π*f_c_*). (8)

In this relation, relaxation frequency is denoted by *f_c_*. In addition, all reported fluctuations are in agreement with the Maxwell–Wagner model [56] as well as with Koop’s approaches [57]. The Maxwell–Wagner bilayer model reveals an increment in hindrance and decrease in conductivity because insulating grain boundary layers isolate the conducting grains of ferrites. Dislocation of charge carriers at void spaces allows electrons to spread at grain boundaries. When grain boundaries possess higher resistances, charges tend to align along boundaries, boosting the polarization process and raising the material’s dielectric characteristics. As the frequency of the ac field rises, the polarization phenomenon is suppressed due to the inability of electrons to follow the rapidly varying frequency path. Such factors significantly interrupt the polarization, ultimately diminishing the dielectric properties. The obtained outcomes are also attributed to plasma treatment. The plasma treatment efficiently reduced hopping probability through grain boundaries, causing an increase in charge accumulation at the side of grain boundaries; consequently, plasma treatment also increases the dielectric permittivity at lower frequency regimes [58]. The parallel trend was profiled for ε′ and ε″ as illustrated in Figure 8a–f.

As a result of the driven ac field, the polarization phenomenon appears and energy is stored in the substance. When an attempt is made to recover that stored energy, a small percentage of it is lost which is termed dissipated energy and can be calculated as [59]:

tan (δ) = ε″/ε′.(9)

Energy dissipation appears as heat, which is the relation between energy gained and lost by the material. Crystal imperfections and charge hopping are two significant causes of such losses. Modifications in the loss tangent (tanδ) and dielectric loss (*ε*″) are illustrated in Figure 8. Equations (10) and (11) were utilized for their calculations.

ε″ = ε′ tanδ(10)

tan (δ) = 1/2 πf R_p_C_p_ (11)

Here, ε″ denotes dielectric loss, R_p_ represents parallel resistance, C_p_ represents parallel capacitance, and tanδ expresses tangent loss. Dielectric loss generated in the material is supported by conduction and relaxation processes due to dipolar impurities in materials. When the frequency is kept low, grain boundaries in the material become highly active, yielding maximum dielectric loss. Additionally, in the high-frequency region, the dielectric loss is initiated by the conduction process of locally restrained charges. Therefore, an interruption is caused in dipole alignment. The loss tangent factor can be affected by homogeneity and degree of defects in the material [60]. A material offering high storage also bears high losses.

A complex impedance modulus is useful for probing the grain boundaries effect. Information about grain and grain boundaries is indispensable for establishing a link between nanostructure and impedance feedback. The electrical impacts of contaminants and grain and grain boundaries existing within a medium can be investigated using impedance analysis. The entire complex impedance comprises real and imaginary components which are related as:

Z* = Z′ + *i*Z″. (12)

**Figure 9:**
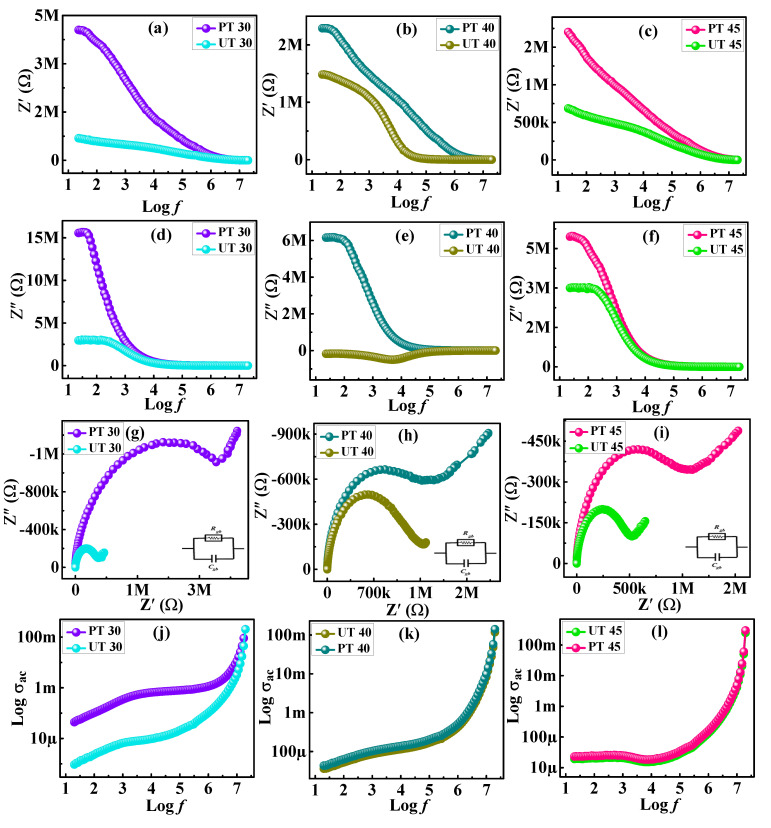
(**a**–**c**) Resistive and (**d**–**f**) reactive responses, (**g**–**i**) Nyquist plot, and (**j**–**l**) ac-conductivity for plasma treated and untreated samples.

In the above equation, Z* denotes complex impedance which contains both real (Z′) and imaginary (Z″) portions. Resistive and reactive responses relative to frequencies are plotted as shown in Figure 9a–f for as-synthesized samples. It is evident from these figures that both graphs profile a declining behavior with increasing frequency. Information about the conductivity of the material is very crucial in explaining the decreasing trend. When the driven ac field was applied at low frequency, then Z′ attained the maximum value while the decline in Z′ values appeared as the frequency approached the high region. According to the Maxwell–Wagner model, such high values of Z′ (low-frequency regimes) may be associated with dominant grain boundaries [61]. The appearance of a relaxation peak in the low-frequency zone confirmed the existence of the relaxation phenomenon. With the steady increment in frequency, the effectiveness of resistive material declined, resulting in shrinkage in the Z′ peak. Additionally, the peak slopes down for plasma-treated ferrite, indicating a lowered impedance. In the same way, Z″ demonstrates a diminishing tendency with increasing frequency [62]. To describe numerous phases of specimens and to generate impedance spectra, Z′ and Z″ were utilized to plot Nyquist plots. Figure 9g–i display the Nyquist plots of pristine and plasma-treated ferrite, plotted between Z′ and Z″ impedance components. Typically, the Nyquist plot consists of semicircular arcs that help examine grain and grain boundary impacts. The arcs obtained as semicircular shapes appeared when bulk resistance was lower than grain boundary resistance. The first arc with a semicircular shape reveals the influence of grain boundaries, while the second and third semicircle arcs denote grain and electrode effects, respectively. Usually, imperfections in grain boundaries cause this because they prevent charge carriers from chasing the changing applied ac field. Charge carriers at grain boundaries display a relaxation process at low frequencies. Due to the greater resistances at low frequencies, they produce semicircular appearances that support the grain boundary effect [63]. Additionally, the semicircle reached higher frequencies, corresponding to the low resistance that the interior grains provide [64]. In the given situation, the semicircular arc discloses only one relaxation phenomenon. Plasma-treated specimens exhibited semicircular arcs with maximum diameters rather than pristine ferrites, which revealed arcs with lower diameters. When the diameter of the arc reduces, it indicates a reduction in resistance while there is an upsurge in conductivity [65]. Additionally, for better investigation of the relaxation process and the Debye-type relaxation process, the formulation of *ac*-conductivity (σ_ac_) was utilized [66]. The conduction process appears due to the motion of charge carriers, transition, grain boundaries, and space charge distribution. All mentioned characteristics take part in total conductivity, which is determined by Jonsher’s power law as demonstrated in Equation (13).

σ _total_ = σ_dc_ + σ_ac_ (13)

In the above equation, σ_dc_ can be determined by using the following equation:

σ_dc_ = d/AB_b_. (14)

Frequency-dependent conductance σ_ac_ can be estimated by

σ_ac_ = εo ε′ω (tanδ). (15)

A boosting conductivity tendency was observed when the frequency was increased spontaneously. When the high frequency is applied, both free and bound charge carriers participate in the conduction process, increasing net conductivity. Examination of the plots between conductivities and frequencies in Figure 9j–i reveals that σ*_ac_* remained the same for some tiny sections at a low frequency, so it can be concluded that it is frequency-independent σ_dc_. Conversely, σ*_ac_* increases towards the high-frequency region. When a supplementary frequency is applied, the exponential growth of σ*_ac_* is accelerated [67]. Furthermore, such conductivity features are based on Koop’s theory which is supported by the Maxwell–Wagner model explaining the idea of grain and grain boundaries [68,69]. The grain boundaries present in the material show large resistance at low frequency, while in the high-frequency region, the material possesses low resistance and, thus conductivity boosts which may be associated with dominant grain effects.

## 4. Conclusions

In summary, in conjunction with the auto-combustion method, the sol–gel process was implemented to synthesize Ni_0.25_Cu_0.25_Zn_0.50_ ferrites. Post synthesis non-thermal plasma treatment of prepared specimens was performed using a microwave source. Structural investigations validated the development of the cubical spinel structure of ferrites. Average crystallite size remained at 49.13 nm for pure and plasma-treated ferrite, but lattice parameters reduced from 8.39 to 8.38 Å and 8.40 to 8.39 Å for plasma-treated specimens. The porosity of the plasma-treated ferrites significantly increased from 5.8 to 7.9% and 6.3 to 7.5% for plasma-treated samples. The morphological study revealed non-uniform grain distribution and dominant agglomeration in pristine ferrites. The average grain size for pristine ferrites was 1.11 μm, which reduced to 0.9 μm for plasma-processed ferrites. *M-H* loops demonstrated a dramatic change in magnetization with plasma treatment. Plasma-treated specimens exhibited 65% greater magnetization than unprocessed ferrites. Maximum saturation magnetization of 74.46 *emu/g*, remnant magnetization of 26.35 *emu/g*, and coercivity of 1040 Oe were observed for plasma-treated specimens with 45 mL solution concentration. A weak ferroelectric behavior was observed for pristine ferrite, which was further interrupted with plasma treatment. Dielectric analysis revealed outstanding enhancement in dielectric characteristics, including dielectric constant, impedance, and σ_ac_ for plasma-treated ferrites. These characteristics highlight the prominence of non-thermal plasma-treated ferrites in cutting-edge devices, which can significantly contribute to energy storage and high-frequency applications.

## Figures and Tables

**Table 1:** The data extracted from XRD spectra of pristine and plasma-processed NiCuZn ferrites.

Sample	Crystallite Size (nm)	Lattice Constant (Å)	Unit Cell Volume	X-ray Density (g/cm^3^)	Bulk Density (g/cm^3^)	Porosity (%)
UT40	49.1212 ± 0.0091	8.3983 ± 0.0081	593.44 ± 1.9242	5.3757 ± 0.0173	5.1732 ± 0.0015	5.881 ± 0.9878
PT40	49.1324	8.3800	593.12	5.3787	5.1723	7.934
UT45	49.1434	8.4001	597.05	5.3433	5.1701	6.321
PT45	49.1201	8.3971	595.99	5.3528	5.1708	7.541

**Table 2:** Measurements of magnetic properties of NiCuZn ferrite samples.

Sample	Saturation Magnetization (*M_s_*)	Remnant Magnetization (*M_r_*)	Squareness Ratio (*M_r_/M_s_*)	Coercivity (*H_c_)*
UT-30	31.85	11.32	0.355	437
PT-30	53.38	18.35	0.343	518
UT-40	42.52	15.31	0.360	443
PT-40	64.48	22.24	0.344	551
UT-45	48.01	20.42	0.425	1033
PT-45	74.46	26.35	0.353	1040

## Data Availability

The reported data is available from the authors on a reasonable request.
